# Supplementary material for: Multiproxy analysis unwraps origin and fabrication biographies of Sardinian figurines: On the trail of metal-driven interaction and mixing practices in the early first millennium BCE
Source: PLoS One. 2025 Sep 10;20(9):e0328268. doi: 10.1371/journal.pone.0328268 (PMC12422461; doi:10.1371/journal.pone.0328268)
Supplement: S1 Appendix — (PDF) [file pone.0328268.s001.pdf]

Multiproxy analysis unwraps origin and fabrication biographies of Sardinian figurines: On the trail of metal-driven interaction and mixing practises in the early first millennium BCE

Daniel Berger, Valentina Matta, Nicola Ialongo, Heide W. Nørgaard, Gianfranca Salis, Michael Brauns, Mads K. Holst, Helle Vandkilde

S1 Appendix. Sample preparation and analytical methods.

Chemical analysis

Chemical analysis was carried out on grinded samples using an ARL Quant'X EDXRF spectrometer (Thermo Fisher Scientific, Bremen, Germany) at the Curt-Engelhorn-Zentrum Archäometrie Mannheim, Germany (CEZA). The spectrometer was equipped with an X-ray tube with a rhodium anode, a Peltier-cooled SD detector and different filters to adjust the X-ray beam to the required excitation conditions. Two excitation settings were used: 28 kV, 900 s and a palladium filter, and 50 kV, 600 s and a copper filter. Calibration was achieved using a set of certified reference copper alloys (BAM-367, BAM-368, ERM-EB374, ERM-EB375, BAM-376) and self-prepared copper alloy in-house references. Quantification followed the empirical method of Lutz and Pernicka [1], which has since been slightly modified. The data was normalised to 100%, and element concentrations are reported in mass% throughout the main paper. Uncertainties of mean values correspond to two standard deviations (2SD).

More sensitive trace element data was obtained by ICP-Q-MS from a selection of samples. For this purpose, small fragments of samples free of corrosion were dissolved overnight in a mixture of 6N HCl and H_2O_2. An aliquot of each solution was analysed, following appropriate dilution, with an XSeries 2 quadrupole mass spectrometer (Thermo Scientific, Bremen, Germany) at CEZA equipped with a collision cell. ^{55}Mn, ^{57}Fe, ^{59}Co, ^{60}Ni, ^{63}Cu, ^{66}Zn, ^{75}As, ^{82}Se, ^{107}Ag, ^{111}Cd, ^{113}In, ^{115}In, ^{118}Sn, ^{121}Sb, ^{126}Te, ^{197}Au, ^{206}Pb and ^{209}Bi were monitored. Calibration was performed using a set of MERCK and SPEX multi- and single-element standard solutions. ^{45}Sc, ^{169}Tm and ^{185}Re, prepared from single stock solutions, were added as internal standards, while spectral interferences on ^{113}In and ^{115}In were corrected using the methodology of Xu et al. [2].

Isotopic analysis

All specimens were dissolved with 6N HCl and H_2O_2 (ca. 20 mg of metal) and aliquots subjected to lead isotope analysis according to a slightly updated protocol by Niederschlag et al. (2003). Part of the sample solutions were also used to determine the tin and copper isotope composition. Isotopic measurements were conducted on a Neptune Plus (Thermo Fisher Scientific, Bremen, Germany) high-resolution multi-collector inductively coupled plasma mass spectrometer (HR-MC-ICP-MS) at CEZA. Details of the sample preparation and the analytical procedure can be found in Brügmann et al. [3] and Berger et al. [4].

The copper isotope values are expressed in delta notation as δ^{65}Cu (= δ^{65}Cu/^{63}Cu) in per mill (‰) relative to the international reference material NIST SRM 976. Analytical uncertainties correspond to two standard deviations (2SD). For tin isotope composition, eight isotope ratios were determined (^{116}Sn/^{120}Sn, ^{117}Sn/^{120}Sn, ^{118}Sn/^{120}Sn, ^{119}Sn/^{120}Sn, ^{122}Sn/^{120}Sn, ^{124}Sn/^{120}Sn, ^{124}Sn/^{116}Sn, ^{117}Sn/^{119}Sn) and referred to NIST SRM 3161a to yield values also in delta notation. However, δSn in per mill per atomic mass unit (‰ u^{-1}), obtained from linear regression analysis with all isotope values, is used for discussion in the main text to facilitate comparison with studies using other isotope values [5–8]. The complete set of isotope values relative to NIST SRM 3161a is given in the Supplementary Material S2 and has been additionally recalculated relative to the Puratronic standard, which was used as an in-house reference material in previous studies [e.g. 9–12]. This recalculation should allow for the best intercomparability of the isotopic compositions determined by different laboratories.

Osmium isotope analyses were carried out at the CEZA following methods described in Brauns [13]. Bronze and copper samples (50 mg metal) were weighed into pre-spiked (^{190}Os tracer) Carius tubes, followed by dissolution and equilibration with inverse aqua regia at 240°C. Osmium was extracted by distillation of its volatile tetra oxide, condensed on a very small volume (20 µl) of chilled H_2SO_4 and then collected in 2 ml of 6.8 N HBr. Final purification of osmium was done by micro-distillation [e.g., 14]. Osmium isotope ratios were measured by ion-counting on a modified Finnigan-MAT 261 operated in NTIMS mode [15,16] and corrected for mass bias and oxides [17]. Internal (2SD) precision for unknowns was $<\pm 0.3\%$. Final ^{187}Os/^{188}Os ratios are corrected for blank (0.05 ± 0.05 pg Os, ^{187}Os/^{188}Os blank 0.108) assuming an osmium yield of 85% [13].

References

1. Lutz J, Pernicka E. Energy dispersive X-ray fluorescence analysis of ancient copper alloys: Empirical values for precision and accuracy. Archaeometry. 1996;38: 313–323.

2. Xu J, Cook NJ, Ciobanu CL, Li X, Kontonikas-Charos A, Gilbert S, et al. Indium distribution in sphalerite from sulfide–oxide–silicate skarn assemblages: a case study of the Dulong Zn–Sn–In deposit, Southwest China. Mineralium Deposita. 2021;56: 307–324. doi:10.1007/s00126-020-00972-y

3. Brügmann G, Berger D, Pernicka E. Determination of the tin stable isotopic composition in tin-bearing metals and minerals by MC-ICP-MS. Geostandards and Geoanalytical Research. 2017;41: 437–448.

4. Berger D, Wang Q, Brügmann G, Lockhoff N, Roberts BW, Pernicka E. The Salcombe metal cargoes: New light on the provenance and circulation of tin and copper in Later Bronze Age Europe provided by trace elements and isotopes. Journal of Archaeological Science. 2022;138: 105543. doi:https://doi.org/10.1016/j.jas.2022.105543

5. Balliana E, Aramendía M, Resano M, Barbante C, Vanhaecke F. Copper and tin isotopic analysis of ancient bronzes for archaeological investigation: Development and validation of a suitable analytical methodology. Analytical and Bioanalytical Chemistry. 2012;405: 2973–2986.

6. Schulze M, Ziegerick M, Horn I, Weyer S, Vogt C. Determination of tin isotope ratios in cassiterite by femtosecond laser ablation multicollector inductively coupled plasma mass spectrometry. Spectrochimica Acta Part B. 2017;130: 26–34.

7. Mason AH, Powell WG, Bankoff HA, Mathur R, Bulatovi A, Filipovi V, et al. Tin isotope characterization of bronze artifacts of the central Balkans. Journal of Archaeological Science. 2016;69: 110–117.

8. Mason AH, Powell WG, Bankoff HA, Mathur R, Price M, Bulatovic A, et al. Provenance of tin in the Late Bronze Age Balkans based on probabilistic and spatial analysis of Sn isotopes. Journal of Archaeological Science. 2020;122: 105181. doi:https://doi.org/10.1016/j.jas.2020.105181

9. Nowell G, Clayton RE, Gale NH, Stos-Gale ZA. Sources of tin: Is isotopic evidence likely to help? In: Bartelheim M, Pernicka E, Krause R, editors. Die Anfänge der Metallurgie in der alten Welt. Rahden/West.: Marie Leidorf; 2002. pp. 291–302.

10. Gillis C, Clayton R. Tin and the Aegean in the Bronze Age. In: Tzachili I, editor. Aegean metallurgy in the Bronze Age Proceedings of an International Symposium held at the University of Crete, Rethymnon, Greece, on November 19-21, 2004. Athens: Ta Pragmata Publications; 2008. pp. 133–142.

11. Haustein M, Gillis C, Pernicka E. Tin isotopy: A new method for solving old questions. Archaeometry. 2010;52: 816–832.

12. Berger D, Figueiredo E, Brügmann G, Pernicka E. Tin isotope fractionation during experimental cassiterite smelting and its implication for tracing the tin sources of prehistoric metal artefacts. Journal of Archaeological Science. 2018;92: 73–86. doi:https://doi.org/10.1016/j.jas.2018.02.006

13. Brauns CM. A rapid, low-blank technique for the extraction of osmium from geological samples. Chemical Geology. 2001;176: 379–384. doi:https://doi.org/10.1016/S0009-2541(00)00371-5

14. Birck JL, Barman MR, Capmas F. Re-Os isotopic measurements at the femtomole level in natural samples. Geostandards Newsletter. 1997;21: 19–27. doi:https://doi.org/10.1111/j.1751-908X.1997.tb00528.x

15. Creaser RA, Papanastassiou DA, Wasserburg GJ. Negative thermal ion mass spectrometry of osmium, rhenium and iridium. Geochimica et Cosmochimica Acta. 1991;55: 397–401. doi:https://doi.org/10.1016/0016-7037(91)90427-7

16. Völkening J, Walczyk T, G. Heumann K. Osmium isotope ratio determinations by negative thermal ionization mass spectrometry. International Journal of Mass Spectrometry and Ion Processes. 1991;105: 147–159. doi:10.1016/0168-1176(91)80077-Z

17. Reisberg L, Meisel T. The Re-Os isotopic system: A review of analytical techniques. Geostandards Newsletter. 2002;26: 249–267. doi:https://doi.org/10.1111/j.1751-908X.2002.tb00633.x
