# Supplementary material for: Multiproxy analysis unwraps origin and fabrication biographies of Sardinian figurines: On the trail of metal-driven interaction and mixing practices in the early first millennium BCE
Source: PLoS One. 2025 Sep 10;20(9):e0328268. doi: 10.1371/journal.pone.0328268 (PMC12422461; doi:10.1371/journal.pone.0328268)
Supplement: S3 Appendix — (PDF) [file pone.0328268.s003.pdf]

Multiproxy analysis unwraps origin and fabrication biographies of Sardinian figurines: On the trail of metal-driven interaction and mixing practises in the early first millennium BCE

Daniel Berger, Valentina Matta, Nicola Ialongo, Heide W. Nørgaard, Gianfranca Salis, Michael Brauns, Mads K. Holst, Helle Vandkilde

S3 Appendix. The question of Cypriot copper for the production of the *bronzetti*.

The dataset presented in the main article '*Multi-proxy analysis unwraps origin and fabrication biographies of Sardinian figurines*' comprising chemical and isotopic results of bronzes is so far the most extensive of Nuragic metalwork. This allows important and new conclusions to be drawn on the local metallurgy of the Late Bronze Age and Early Iron Age, but provides also the opportunity to review and re-assess aspects raised in earlier stages of archaeometallurgical research. One of these aspects concerns the question of why Cypriot copper was apparently not used for the production of Sardinia bronzes [1–3]. In fact, that is a fair question, particularly when envisaging the large corpus of oxhide and plano-convex ingots (bun ingots) from the island with isotopic and chemical signatures pointing to an origin in Cyprus [3–9]. Admittedly, this aspect has been discussed for already four decades, and a rejection of Cypriot copper by the Nuragic metalworkers seems to be widely accepted today [2,3,7,10]. Nevertheless, the original questions are still not fully answered: "Why are the Sardinians importing a metal which is available in their country?" [2] and "What happened to all the Cypriot copper exported in the form of copper oxhide ingots?" [1].

In 2001, V. Kassianidou addressed both questions while criticising the bold statement of colleagues that Cypriot copper was imported as ingots to but never recast as objects in Bronze Age Sardinia. One possible explanation for the invisibility of Cypriot copper in Sardinian metalwork in Kassianidou's eyes was the mixing of Cypriot with local copper (leaded copper), lead or tin, explaining the high concentrations of lead and the consequential masking of the Cypriot lead isotope signature. Arguing along similar lines, though beyond the geographical scope of interest here, Bruyère et al. [11] propose that Cypriot copper was imported to the Eastern Balkans as oxhide ingots (some of which have survived) and used for local metal production, but remained undetected due to the overprinting of its isotopic signature. Given the in general very low concentrations of lead in oxhide or bun ingots – e.g. 0.013% on average for

Sardinian oxhide ingots (n = 49) or 0.034% for the Uluburun oxhide ingots (n = 52) [3,6,12] – opposed to the omnipresent lead in Sardinian ores [13], this hypothesis is reasonable, not least because Cypriot copper could not be cast without alloying. In a late reply, Gale [7] referred to the idea of source mixing and was confident of being able to disprove Kassianidou's reasoning on the basis of the chemical composition of ingots and bronzes. Likewise, Begemann et al. [3], slightly earlier than Kassianidou [2], argued against the mixing of ingot copper with local sources of Sardinia.

Methodological developments have now produced fine-grained and good quality data in sufficient amounts. This invites a revitalisation of the discussion 'Cypriot/Sardinian' aiming to answer the question whether Cypriot copper was the source behind the *bronzetti* sampled for this study. Admittedly, the objects in question are younger than the majority of oxhide ingots, but some late copper from Cyprus could still have been in circulation at the time when the figurines were produced.

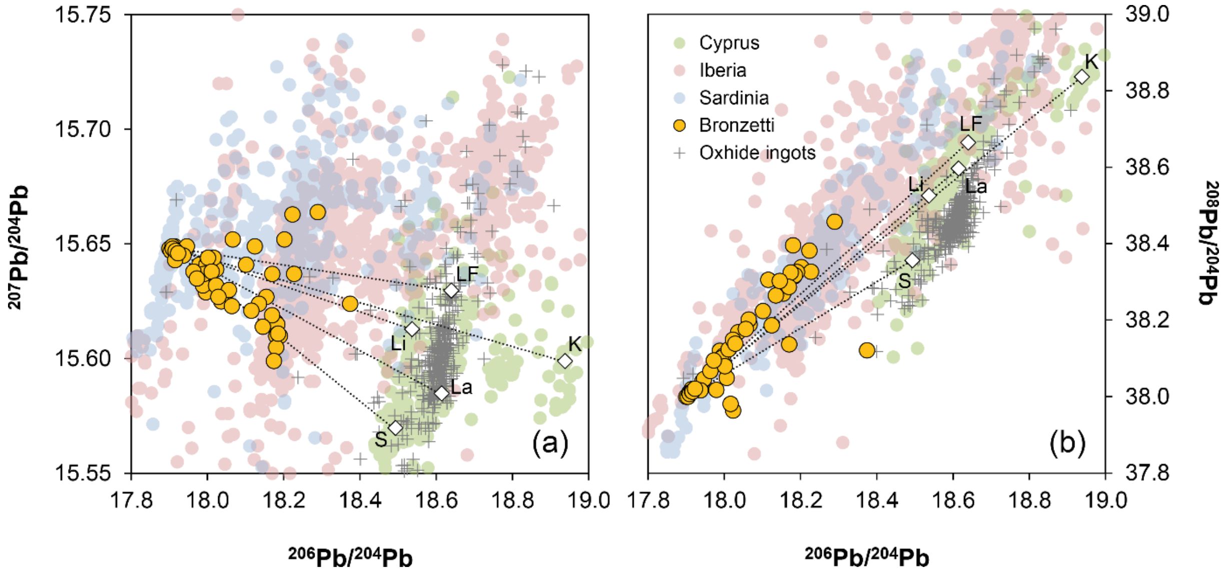

Fig S3.1. Lead isotope ratios of the studied figurines compared with those of copper and lead ores from Sardinia, Iberia, and Cyprus. The dotted lines represent mixing lines, which are expected if there was mixing between copper from Cu1 copper pool and Cypriot ores from the Solea (S), Larnaca (La), Limni (Li), Limmasol Forest (LF), and Kalavassos (K) axes. The data points of Cypriot deposits (white diamonds) correspond to the mean isotope ratios for all ores within the respective regions (diagrams: D Berger; data from ingots and ores from references given in S4 Appendix).

Figure S3.1 shows that the lead isotope composition of the bronzes and oxhide/bun ingots are completely different. While the lead isotope ratios of the ingots point to sources in Cyprus, the lead isotopes of the figurines are compatible with Sardinian or Iberian ores. Thus, a direct production of the bronzes from the ingots is impossible. However, as hypothesised in the main article, part of the bronzes was most likely made by source mixing, which opens the possibility of a contribution of oxhide/bun ingots to the isotopic signatures of the bronzes. In fact, in the diagram Figure S3.1a, the extension of the presumed mixing line of the Santa Vittoria bronzes (ML1) perfectly meets the mean isotopic composition of copper ores from the Cypriot Solea area, where the Apliki mine as the most likely supplier for the oxhide and bun ingots is situated [4,7,14]. The compositional pattern of ores from other regions on Cyprus is less plausible in terms of the Santa Vittoria bronze objects, whereas the average of ores from the Limassol forest area accords well with the three *bronzetti* samples from Abini-Teti. In contrast, no relationship would exist for the bronzes from Su Monte. Thus, based on the $^{206}Pb/^{204}Pb$–$^{207}Pb/^{204}Pb$ diagram, a relationship between *bronzetti* and Cypriot ores would be feasible. Yet, this is just one part of the story. When accounting for the $^{208}Pb/^{204}Pb$ ratios (Fig. S3.1b), none of the means of the Cypriot ores is in line with the bronzes, which would be expected if there were a mixture with them. Only in case of very large differences in the lead concentrations of the original sources for the bronzework, a scenario of no clear mixing line would evolve, and the Cypriot signature would have been overprinted completely. Given the high lead concentrations of the present bronzes (e.g. average of the Santa Vittoria bronzes (XRF): 1.3% Pb) compared to the very low in the ingots (0.013/0.034%; Begemann et al., 2001; Hauptmann et al., 2002), this explanation could apply. However, this should then be true for all combinations of isotope ratios, which is obviously not the case. In addition, the figurines closer to the data points of the ores from Cyprus in the $^{206}Pb/^{204}Pb$ vs. $^{207}Pb/^{204}Pb$ diagram are richer in lead than those farther away potentially representing one end member of the suspected mixing line. In conclusion, one would deduce from this observation that the extended mixing line, including the Solea area mean, is only accidental and that there is no direct relationship between the ores from Cyprus and the *bronzetti*. At the same time, however, a relationship is still possible even if the Cypriot signature is obscured, but then one had to suggest a mixture of three components: two to establish the Santa Vittoria mixing line and a third one being the oxhide/bun ingots not visible in the lead isotope data.

A definite decision about the original questions is thus not possible without a combined consideration of the chemical composition, particularly the trace elements of both the bronzes

and the ingots. Clearly, the Nuragic bronzework analysed in this paper is by one to two magnitudes of order richer in lead, silver and antimony than the oxhide ingots, while cobalt, nickel, arsenic, and bismuth are similar (Fig. S3.2). If one assumes mixing of materials following the above reasoning, there are several hypothetical combinations depending on whether two or three pools of materials were mixed:

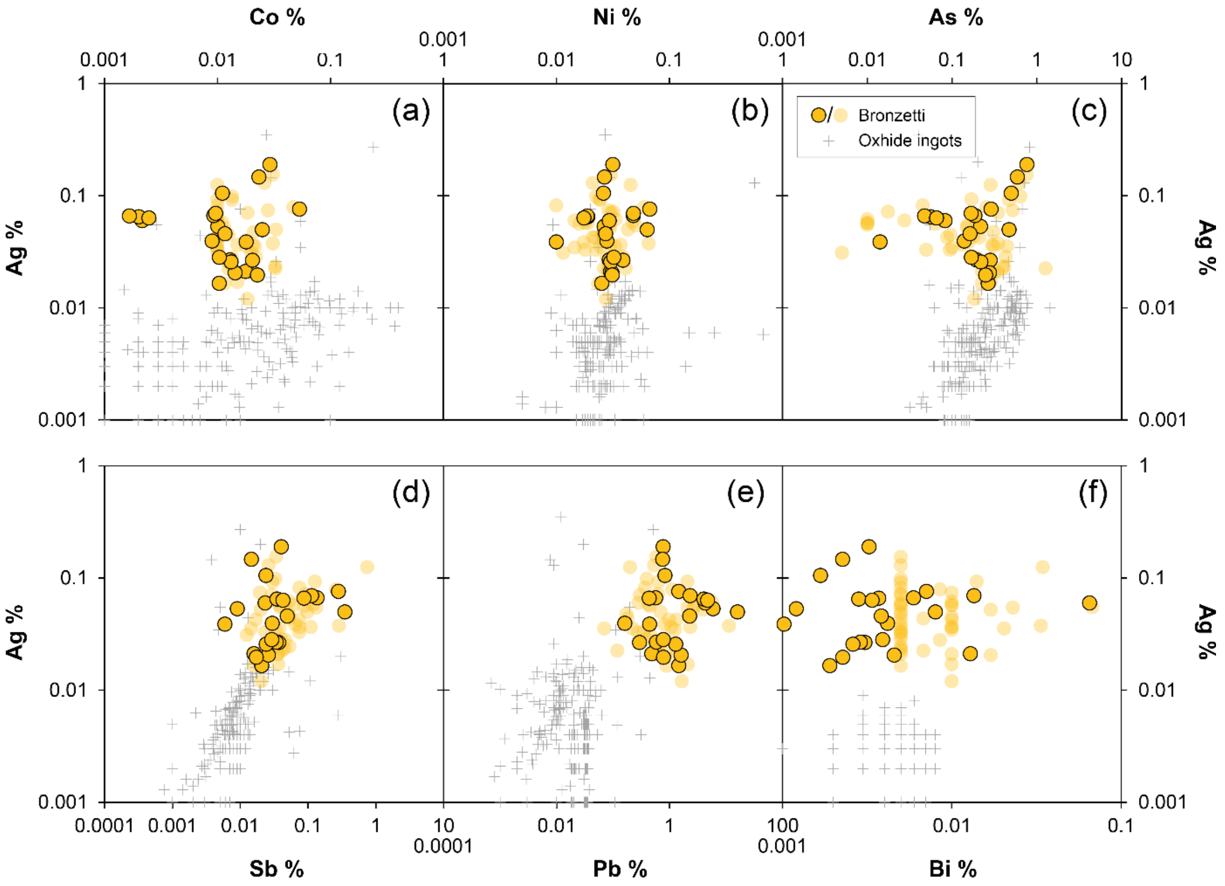

Fig 12. Elemental composition of the analysed figurines compared with that of oxhide and bun ingots of Cypriot copper. All concentrations in mass% (diagrams: D Berger; data from ingots and ores from references given in S4 Appendix).

1. Two materials

- Cypriot copper + Sardinian lead
- Cypriot copper + Sardinian copper containing natural impurities of lead

2. Three materials

- Cypriot copper + Sardinian copper intentionally alloyed with Sardinian lead (leaded copper)
- Cypriot copper + Sardinian copper + foreign lead
- Cypriot copper + Sardinian copper containing natural impurities of lead + foreign copper containing natural impurities of lead

Let us first consider the simplest case in which oxhide ingots were alloyed with Sardinian lead. This option was put forward by Kassianidou [2], who tried to explain the blatant absence of Cypriot copper in Sardinian Bronze Age bronzes while challenged by Gale [7]. If we assume the ca. 5% lead in two of the Santa Vittoria *bronzetti* (MA-202529, MA-202533) as deliberate addition of Sardinian lead and take the average silver concentration of 0.009±0.015% in Nuragic lead objects [6,13,15,16] as a basis, then a rather equal amount of silver would have mixed with the on average 0.011±0.043 % silver in oxhide ingots found in Sardinia [3,6] or the 0.004±0.009% in the Uluburun oxhide ingots [12]. The silver concentration in an alloying scenario with Sardinian lead would consequently have increased the silver of Cypriot copper very little, and certainly not by a factor of ten (see eq. 2; Fig. S3.2e), an effect that would have been even less pronounced if we consider the average lead content of 1.3% of all *bronzetti* from Santa Vittoria instead of 5%.

This conclusion is not substantially different if we use the limited chemical data available for Sardinian galenas, as the silver concentrations are similar or slightly higher [17–19]. Similar constraints apply to other elements such as antimony and bismuth, which are contained in Sardinian lead and ores in concentrations below 0.01% or even lower [15–17,19]. Given the data is representative of Sardinian lead and lead ores, and no other material than these sources have been used to make leaded copper/bronze, the *bronzetti*'s elemental pattern can be excluded to result from mixing or alloying of oxhide ingots with lead.

The situation can be a different one if galena of other regions were used. Depending on the geography and geology, lead ores can contain silver, antimony and bismuth of up to 0.1% or often higher [e.g. 20–25]. For example, in galena from zinc-lead deposits in the Alcudia Valley, Spain, silver and antimony contents of more than 0.3 and 0.7% were observed [21]. Using such galena could produce lead metal with elevated trace element concentrations, which in turn would have had a noticeable effect on the composition of the mixture. If we consider again the

above example of the *bronzetti* with 5% lead, this time with 0.3% silver, the silver concentration in a leaded copper would calculate to [26]:

$$X_{mixture} = X_{ingot} \times f_{ingot} + X_{lead} \times (1 - f_{ingot}) \quad \text{(eq. 1)}$$

where X is the concentration of silver in the mixture and the raw materials and f the mass fraction of the raw materials. Using the above figures from example 1 and 2 yield

$$X_{mixture} = 0.011 \times 0.95 + 0.009 \times 0.05 \ \ [\%] \quad \text{(eq. 2)}$$
$$X_{mixture} = 0.0109\%$$

and

$$X_{mixture} = 0.011 \times 0.95 + 0.3 \times 0.05 \ \ [\%] \quad \text{(eq. 3)}$$
$$X_{mixture} = 0.0254\%$$

Equation 3 clearly shows that the concentration of silver in copper is only moderately increased (and so is Sb), even if the metal is alloyed with 0.3% silver from the lead (or galena). To raise the trace composition of silver in the copper by a factor of ten (to 0.1%) would therefore require lead with about 1.8 % silver, or more if the ingot's silver content was lower or less lead was used for mixing. Since such high silver contents in galena are very rare, we can exclude a mixture of oxhide ingot copper and lead, still provided only two materials were used. Nevertheless, the lead isotope signature of the ingots is altered and obscured drastically in this mixing scenario because of an abundance of lead in the bronzes even if the chemistry is not.

The isotopic composition will have changed in the same way when ingot copper was mixed with another copper base containing elevated natural impurities of lead. In this case, also the chemical composition can change significantly, depending on the content of trace elements and the proportions of the copper bases. As an example, we use Sardinian copper minerals/ores and the typical ranges of their impurities (Co, Ni, As, Ag, Sb, Pb), even though only a limited amount of data currently exists. The study of Begemann et al. [3] is the only one having reported useful data of copper ores so far, probably because Sardinia is primarily known for its abundant zinc and lead deposits with elevated silver grades rather than for its copper sources [13,18,27–29]. Nevertheless, mineralisation often have polymetallic character, and enrichment of copper

is frequently observed, especially in the mines of Funtana Raminosa, Sa Duchessa and Calabona (Fig. 2) [4,13]. Generally, the chemical compositions of copper ores are quite divers, depending on the region and geological environment from which the material derives. Typical contents of arsenic, antimony, nickel, silver and lead are in the range of several hundred µg g^{-1}, but can easily reach concentrations of one to several per cent when normalised to the copper content [3, Table 6]. It is easy to understand that the chemical composition of a low impurity copper base (such as the oxhide ingot copper) is changed considerably when mixed with copper from such copper ores, even when losses during the pyrometallurgical *chaîne opératoire* are considered [30]. To raise for example the silver or any other element concentration by a factor of ten from 0.01 to 0.1% only 0.185% of the respective element in copper is needed in case of a 1:1 mixture.

In conclusion, it appears to be quite easy to alter the chemical signature of Cypriot copper when it is mixed with copper from Sardinia. However, in theory, mixing lines may evolve for pairs of elements in their mixing products, which is when objects as mixtures of different proportions of these raw materials exist. This can provide possibilities for the recognition of mixing copper from Cyprus and Sardinia or somewhere else. However, in the present context, no mixing lines show up in the elemental diagrams (Fig. S3.2) relatable to oxhide ingots. Rather the bronzes exhibit both positive and negative correlations among each other, as shown in the main document, especially for arsenic with antimony and silver with different elements. It has to be stressed though that positive correlations must not *per se* be indicative of blending different materials. Correlations in a pool of copper-based artefacts can also appear if for example specific minerals in the ore charges were smelted, such as gersdorffite (NiAsS) or fahlores, resulting in correlations of nickel and arsenic or arsenic, antimony, bismuth and silver [31,32]. Conversely, negative correlations are much better evidence of intentional mixing since paragenetic reasons in the ores can be excluded. Because the latter situation is observed within the studied figurines, mixing of two materials is likely. However, as the mixing lines do not involve the data of the copper ingots, it is again very unlikely that Cypriot copper played a role in the production process of the *bronzetti*.

At this point, we could continue to argue about the effects of more than two materials on the chemical and lead isotope composition to illustrate ways in which Cypriot copper could still be a source of the bronzes. However, we will not do so for the following reasons: First, a deliberate simultaneous mixing of three raw materials at the same place – in particular three copper bases

– with three different origins is unlikely. It is more reasonable that this occurred sequentially, i.e. by mixing of two bases at one place first and a later additional mixing with a third one at a different location. This could also involve recycling. In this case, the considerations from above would still apply as it is a two-step mixing procedure of two materials each step (cf. Fig. 13 in main document). Secondly, the argumentation would become even more theoretical and difficult to follow if three material bases were taken into account, which would necessitate introducing mathematic mixing models. However, this would go beyond the scope of this paper.

References

1. Muhly JD. The significance of metals m the Late Bronze Age economy of Cyprus. In: Karageorghis V, Michaēlidēs D, editors. The development of the Cypriot economy: From the prehistoric period to the present day. Nicosia: University of Cyprus; 1996. pp. 45–60. Available: https://books.google.de/books?id=F8zsAAAAMAAJ

2. Kassianidou V. Cypriot copper in Sardinia. Yet another case of bringing coals to Newcastle?, in: Bonfante, L., Karageorghis, V. (eds.), pp. 97–119. In: Bonfante L, Karageorghis V, editors. Italy and Cyprus in antiquity: 1500–450 BC Proceedings of an International Symposium held at the Italian Academy for Advanced Studies in America at Columbia University, November 16–18, 2000. Nicosia: The Costakis and Leto Severis Foundation; 2001. pp. 97–119.

3. Begemann F, Schmitt-Strecker S, Pernicka E, Lo Schiavo F. Chemical composition and lead isotopy of copper and bronze from Nuragic Sardinia. European Journal of Archaeology. 2001;4: 43–85.

4. Gale NH, Stos-Gale ZA. Oxhide ingots from Sardinia, Crete and Cyprus and the Bronze Age copper trade: New scientific evidence. In: Balmuth MS, editor. Studies in Sardinian archaeology III: Nuragic Sardinia and the Mycenaean World. Oxford: Oxbow; 1987. pp. 135–178.

5. Gale NH, Stos-Gale ZA. Recent evidence for a possible Bronze Age metal trade between Sardinia and the Aegean. In: French ER, Wardle KA, editors. Problems in Greek prehistory: Papers presented at the Centenary Conference of the British School of Archaeology at Athens, Manchester, April 1986. Bristol: Bristol Classical Press; 1988. pp. 349–383. Available: https://books.google.de/books?id=30hoAAAAMAAJ

6. Atzeni C, Massidda L, Sanna U. Investigations and results. In: Lo Schiavo F, Giumlia-Mair A, Sanna U, Valera R, editors. Archaeometallurgy in Sardinia from the origin to the Early Iron Age. Montagnac: M. Mergoil; 2005. pp. 115–183.

7. Gale NH. Lead isotope studies: Sardinia and the Mediterranean. Provenance studies of artefacts found in Sardinia. Instrumentum. 2006;23: 29–34.

8. Hauptmann A. Lead isotope analysis and the origin of sardinian metal objects. In: Lo Schiavo F, Muhly JD, Maddin R, Giumlia-Mair A, editors. Oxhide ingots in the central Mediterranean. Roma: A.G. Leventis Foundation ; CNR-Istituto di studi sulle civiltà dell'Egeo e del vicino oriente; 2009. pp. 500–514.

9. Sabatini S, Lo Schiavo F. Late Bronze Age metal exploitation and trade. Materials and Manufacturing Processes. 2020. Available: https://doi.org/10.1080/10426914.2020.1758329

10. Kassianidou V. The production, use and trade of metals in Cyprus and Sardinia: So similar and yet so different. Instrumentum. 2006;23: 34–37.

11. Bruyère C, Daly JS, Acken D van, Jovanović D, Orfanou V, Franković F, et al. Trade, recycling and mixing in local metal management strategies of the later Bronze Age south Carpathian Basin: Lead isotope and chemical analyses of hoarded metalwork. Journal of Archaeological Science. 2024;164: 105957. doi:https://doi.org/10.1016/j.jas.2024.105957

12. Hauptmann A, Maddin R, Prange M. On the structure and composition of copper and tin ingots excavated from the shipwreck of Uluburun. Bulletin of the American Schools of Oriental Research. 2002. pp. 1–30.

13. Valera RG, Valera PG, Rivoldini A. Sardinian ore deposits and metals in the Bronze Age. In: Lo Schiavo F, Giumlia-Mair A, Sanna U, Valera R, editors. Archaeometallurgy in Sardinia from the origin to the Early Iron Age. Montagnac: M. Mergoil; 2005. pp. 43–87.

14. Gale NH, Stos-Gale ZA. The role of Apliki mine region in the post c.1400 BC copper production and trade networks in Cyprus and in the wider Mediterranean. In: Kassianidou V, Papasavvas G, editors. Eastern Mediterranean metallurgy and metalwork in the second millennium BC: A conference in honour of James D Muhly, Nicosia, 10th-11th October 2009. Oxford: Oxbow Books; 2012. pp. 70–82.

15. Atzeni C, Massidda L, Sanna U, Virdis P. Notes on lead metallurgy in Sardinia during the Nuragic period. Historial Metallurgy. 1990;24: 97–106.

16. Cincotti A, Massidda L, Sanna U. Chemical and isotope characterization of lead finds at the Santa Barbara nuraghe (Bauladu, Sardinia). Journal of Cultural Heritage. 2003;4: 263–268. doi:https://doi.org/10.1016/j.culher.2003.06.002

17. Novello A, Schena G, Censi P, Speziale S, Comin-Chiaramonti P. Distribuzione di elementi in traccia nei giacimenti a solfuri misti della Sardegna: uno studio pilota. Mineralogica et petrographica acta. 1997;40: 257–269.

18. Moroni M, Naitza S, Ruggieri G, Aquino A, Costagliola P, Giudici GD, et al. The Pb-Zn-Ag vein system at Montevecchio-Ingurtosu, southwestern Sardinia, Italy: A summary of previous knowledge and new mineralogical, fluid inclusion, and isotopic data. Ore Geology Reviews. 2019;115: 103194. doi:https://doi.org/10.1016/j.oregeorev.2019.103194

19. Albarède F, Boni M, Blichert-Toft J, Vaxevanopoulos M, Westner K, Milot J, et al. Ag and Pb isotope systematics in galena ores from southern Sardinia and southern France flag potential silver sources in antiquity. Archaeological and Anthropological Sciences. 2024;16: 118. doi:10.1007/s12520-024-02025-1

20. Zhang Q. Trace elements in galena and sphalerite and their geochemical significance in distinguishing the genetic types of Pb-Zn ore deposits. Geochemistry. 1987;6: 177–190.

21. Palero-Fernández FJ, Martín-Izard A. Trace element contents in galena and sphalerite from ore deposits of the Alcudia Valley mineral field (Eastern Sierra Morena, Spain). Journal of Geochemical Exploration. 2005;86: 1–25.

22. George L, Cook NJ, Ciobanu CL, Wade BP. Trace and minor elements in galena. American Mineralogist. 2015;100: 548–569.

23. Milot J, Blichert-Toft J, Sanz MA, Malod-Dognin C, Télouk P, Albarède F. Silver isotope and volatile trace element systematics in galena samples from the Iberian Peninsula and the quest for silver sources of Roman coinage. Geology. 2022;50: 422–426. doi:10.1130/G49690.1

24. Vaxevanopoulos M, Davis G, Milot J, Blichert-Toft J, Malod-Dognin C, Albarède F. Narrowing provenance for ancient Greek silver coins using Ag isotopes and Sb contents of potential ores. Journal of Archaeological Science. 2022;145: 105645. doi:https://doi.org/10.1016/j.jas.2022.105645

25. Aldis C, Olivo GR, Morfin S. LA-ICP-MS trace element composition of sphalerite and galena of the proterozoic carbonate-hosted Morro Agudo Zn-Pb sulfide district, Brazil: Insights into ore genesis. Minerals. 2022;12. doi:10.3390/min12081028

26. Faure G, Mensing TM. Isotopes: Principles and applications. 3. Hoboken: John Wiley & Sons; 2005.

27. Arce M, Tuveri C. Nota sulle miniere di rame in Sardegna. 1. ed. In: Kirova TK, editor. L'uomo e le miniere in Sardegna. 1. ed. Cagliari: Edizioni Della Torre; 1993. pp. 21–23.

28. Giardino C. Il Mediterraneo Occidentale fra XIV ed VIII secolo a.C. Cercie minerarie e metallurgiche / The West Mediterranean between the 14th and 8th Centuries B.C. Oxford: Tempus Reparatum; 1995. doi:10.30861/9780860547938

29. Pearce M. The 'island of silver veins': an overview of the earliest metal and metalworking in Sardinia. Metalla. 2017;23: 91–111.

30. Tylecote RF, Ghaznavi HA, Boydell PJ. Partitioning of trace elements between the ores, fluxes, slags and metal during the smelting of copper. Journal of Archaeological Science. 1977;4: 305–333. doi:https://doi.org/10.1016/0305-4403(77)90027-9

31. Pernicka E, Lutz J, Stöllner T. Bronze Age copper produced at Mitterberg, Austria, and its distribution. Archaeologia Austriaca. 2016;100: 19–55.

32. Dardeniz G. Why did the use of antimony-bearing alloys in Bronze Age Anatolia fall dormant after the Early Bronze Age?: A case from Resuloğlu (Çorum, Turkey). PLOS ONE. 2020;15: 1–34. doi:10.1371/journal.pone.0234563
