# Supplementary material for: Multiproxy analysis unwraps origin and fabrication biographies of Sardinian figurines: On the trail of metal-driven interaction and mixing practices in the early first millennium BCE
Source: PLoS One. 2025 Sep 10;20(9):e0328268. doi: 10.1371/journal.pone.0328268 (PMC12422461; doi:10.1371/journal.pone.0328268)
Supplement: S4 Appendix — (PDF) [file pone.0328268.s004.pdf]

Multiproxy analysis unwraps origin and fabrication biographies of Sardinian figurines: On the trail of metal-driven interaction and mixing practises in the early first millennium BCE

Daniel Berger, Valentina Matta, Nicola Ialongo, Heide W. Nørgaard, Gianfranca Salis, Michael Brauns, Mads K. Holst, Helle Vandkilde

S4 Appendix. Additional references.

Ores

Cyprus: [1]

Iberian peninsula: [2–4] [5] [6] [7–10] [11–29] [3,30,31]

Sardinia: [3,32–37]

Arabah: [38–44]

Artefacts

Oxhide ingots: [32,35,45–48]

Bronze artefacts and figurines: [32,45,47,49–51]

Lead artefacts: [35,45,52,53]

References

1. Gale NH, Stos-Gale ZA, Maliotis G, Annetts N. Lead isotope data from the Isotrace Laboratory, Oxford: Archaeometry data base 4, ores from Cyprus. Archaeometry. 1997;39: 237–246.

2. Arribas A, Tosdal RM. Isotopic composition of Pb in ore deposits of the Betic Cordillera, Spain: Origin and relationship to other European deposits. Economic Geology. 1994;89: 1074–1093. doi:10.2113/gsecongeo.89.5.1074

3. Stos-Gale ZA, Gale NH, Houghton J, Speakman R. Lead isotope data from the Isotrace Laboratory, Oxford: Archaeometry Data Base 1, ores from the Western Mediterranean. Archaeometry. 1995;37: 407–415.

4. Murillo-Barroso M, Montero Ruiz I, Nieto JM, Camalich Massieu MD, Martín Socas D, Martinón-Torres M. Trace elements and lead isotopic composition of copper deposits from the eastern part of the Internal Zone of the Betic Cordillera (SE Iberia): application to provenance of

archaeological materials. Journal of Iberian Geology. 2019;45: 585–608. doi:10.1007/s41513-019-00111-1

5. Bartelheim M, Cortés FC, Onorato AM, Murillo-Barroso M, Pernicka E. The silver of the South Iberian El Argar Culture: A first look at production and distribution. Trabajos de prehistoria. 2012;69: 293–309.

6. Montero Ruiz I, Murillo-Barroso M. La producción metalúrgica en las sociedades argáricas y sus implicaciones sociales: Una propuesta de investigación. Menga: Revista de Prehistoria de Andalucía. 2010;1: 37–52.

7. Baron S, Rico C, Antolinos Marín JA. Le complexe d'ateliers du Cabezo del Pino (Sierra Minera de Cartagena-La Unión, Murcia) et l'organisation de l'activité minière à Carthago Noua à la fin de la République romaine: Apports croisés de l'archéologie et de la géochimie. Archivo Español de Arqueología. 2017;90: 147–169. doi:10.3989/aespa.090.017.007

8. Dayton JE, Dayton A. Uses and limitations of lead isotopes in archaeology. In: Olin JS, Blackman MJ, editors. Washington, D. C.; 1986. pp. 13–41.

9. Graeser S, Friedrich G. Zur Frage der Altersstellung und Genese der Blei-Zink-Vorkommen der Sierra de Cartagena in Spanien. Mineral Deposita. 1970;5: 365–374. doi:10.1007/BF00206733

10. Klein S, Domergue C, Lahaye Y, Brey GP, Kaenel H-M von. The lead and copper isotopic composition of copper ores from the Sierra Morena (Spain). Journal of Iberian Geology. 2009;35: 59–68.

11. Arias D, Corretge LG, Suarez O, Villa L, Cuesta A, Gallastegui G. Lead and sulfur isotope compositions of the Ibias gold vein system (NW Spain); genetic implications. Economic Geology. 1996;91: 1292–1297. doi:10.2113/gsecongeo.91.7.1292

12. García de Madinabeitia S. Implementación y aplicación de los análisis isotópicos de Pb al estudio de las mineralizaciones y la geocronología del área Los Pedroches-Alcudia (Zona Centro-Ibérica). Universidad del País Vasco. 2003.

13. García de Madinabeitia S, Ibarguchi JIG, Zalduegui JFS. IBERLID: A lead isotope database and tool for metal provenance and ore deposits research. Ore Geology Reviews. 2021;137: 104279. doi:https://doi.org/10.1016/j.oregeorev.2021.104279

14. Hermanns MH. La Zona Minera De S'Argentera, Isla De Ibiza (Islas Baleares). Cuadernos de Prehistoria y Arqueología de la Universidad de Granada. 2014;24: 301–318.

15. Huelga-Suarez G, Moldovan M, Suárez Fernández M, De Blas Cortina MÁ, García Alonso JI. Isotopic composition of lead in copper ores and a copper artefact from the La Profunda Mine (León, Spain). Archaeometry. 2014;56: 651–664.

16. Huelga-Suarez G, Moldovan M, Suárez Fernández M, De Blas Cortina MÁDB, Garciá Alonso JI. Defining the lead isotopic fingerprint of copper ores from North-West Spain. Archaeometry. 2014;56: 88–101.

17. Huelga-Suarez G, Moldovan M, Suárez Fernández M, De Blas Cortina MÁ, Vanhaecke F, García Alonso JI. Lead isotopic analysis of copper ores from the Sierra El Aramo (Asturias, Spain). Archaeometry. 2012;54: 685–697. doi:10.1111/j.1475-4754.2011.00635.x

18. Hunt Ortiz MA, Llull Estrellas B, Perelló Mateo L, Salvà Simonet B. Aprovechamiento de recursos cupríferos en la edad del bronce de Menorca: La Mina se Sa Mitja Lluna (Illa Den Colom). Cuadernos de Prehistoria y Arqueología de la Universidad de Granada. 2014;24: 45–109.

19. Ibáñez Gómez JA. Estudio metalogenético de las mineralizaciones de plomo, zinc y cobre en el paleozoico de la Sierra de La Demanda (La Rioja-Burgos). Universidad del País Vasco. 1998.

20. Jaramillo Justinico A. Recursos y materias primas en la Edad del Bronce del Alto Guadalquivir, medioambiente y registro arqueológico en la cuenca del río Rumblar. Universidad de Granada. 2005. Available: http://hdl.handle.net/10481/712

21. Medina J, Tassinari C, Martins MER, Kawashita K, Azevedo MR, Santos JF, et al. Mineralizações de galenas em Portugal: composição isotópica do chumbo. In: Ferreira MPV, editor. A geologia de engenharia e os recursos geológicos Vol 2: Recursos geológicos e formação. Coimbra: Imprensa da Universidade de Coimbra; 2003. pp. 169–178. doi:10.14195/978-989-26-0322-3_12

22. Perelló Mateo L. Tecnología metalúrgica del cobre y del bronce durante el período ostalayótico en Mallorca (ca. s. VI a. C. - s. I a. C.). Universitat de les Illes Balears. 2017. Available: https://dspace.uib.es/xmlui/handle/11201/148980

23. Renzi M, Bode M, Marzoli D, Aguayo de Hoyos P, León Martín C, Sierra de Cózar G, et al. Ausbeutung von Bergbauressourcen im Umland von Los Castillejos de Alcorrín (Manilva, Málaga) (Ende 9. und 8. Jh. v. Chr.) : ein Vorbericht. Madrider Mitteilungen. 2016;57: 139–211. doi:10.34780/MM.V57I0.1004

24. Sáez R, Nocete F, Gil Ibarguchi JI, Rodríguez-Bayona M, Inacio N, Quispe D, et al. A lead isotope database for copper mineralization along the Guadalquivir River Valley and surrounding areas. Journal of Iberian Geology. 2021;47: 411–427. doi:10.1007/s41513-020-00151-y

25. Santos Zalduegui JF, Garcia De Madinabeitia S, Gil Ibarguchi JI, Palero F. A lead isotope database: The Los Pedroches-Alcudia area (Spain): Implications for archaeometallurgical connections across southwestern and southeastern Iberia. Archaeometry. 2004;46: 625–634. doi:10.1111/j.1475-4754.2004.00178.x

26. Soares AMM, Valério P, Gomes SS, Mataloto R, Soares SM, Silva RJC, et al. A first appraisal on copper sources for Chalcolithic settlements in southern Portugal using Pb isotope analysis. Journal of Archaeological Science: Reports. 2020;33: 102481. doi:10.1016/j.jasrep.2020.102481

27. Subías I, Fanlo I, Mateo E, Billström K, Recio C. Isotopic studies of Pb–Zn–(Ag) and barite Alpine vein deposits in the Iberian Range (NE Spain). Geochemistry. 2010;70: 149–158. doi:10.1016/j.chemer.2009.12.004

28. Tornos F, Chiaradia M. Plumbotectonic Evolution of the Ossa Morena Zone, Iberian Peninsula:Tracing the Influence of Mantle-Crust Interaction in Ore-Forming Processes. Economic Geology. 2004;99: 965–985. doi:10.2113/gsecongeo.99.5.965

29. Velasco F, Pesquera A, Herrero JM. Lead isotope study of Zn-Pb ore deposits associated with the Basque-Cantabrian basin and Paleozoic basement, Northern Spain. Mineralium Deposita. 1996;31: 84–92.

30. Marcoux E. Lead isotope systematics of the giant massive sulphide deposits in the Iberian Pyrite Belt. Mineralium Deposita. 1997;33: 45–58.

31. Pomiès C, Cocherie A, Guerrot C, Marcoux E, Lancelot J. Assessment of the precision and accuracy of lead-isotope ratios measured by TIMS for geochemical applications: example of massive sulphide deposits (Rio Tinto, Spain). Chemical Geology. 1998;144: 137–149. doi:10.1016/S0009-2541(97)00127-7

32. Begemann F, Schmitt-Strecker S, Pernicka E, Lo Schiavo F. Chemical composition and lead isotopy of copper and bronze from Nuragic Sardinia. European Journal of Archaeology. 2001;4: 43–85.

33. Boni M, Koeppel V. Ore-lead isotope pattern from the Iglesiente-Sulcis Area (SW Sardinia) and the problem of remobilization of metals. Mineralium Deposita. 1985;20: 185–193. doi:10.1007/BF00204563

34. Caron C, Lancelot J, Omenetto P, Orgeval J-J. Role of the Sardic tectonic phase in the metallogenesis of SW Sardinia (Iglesiente): lead isotope evidence. ejm. 1997;9: 1005–1016. doi:10.1127/ejm/9/5/1005

35. Gale NH, Stos-Gale ZA. Oxhide ingots from Sardinia, Crete and Cyprus and the Bronze Age copper trade: New scientific evidence. In: Balmuth MS, editor. Studies in Sardinian archaeology III: Nuragic Sardinia and the Mycenaean World. Oxford: Oxbow; 1987. pp. 135–178.

36. Ludwig KR, Vollmer R, Turi B, Simmons KR, Perna G. Isotopic constraints on the genesis of base-metal ores in southern and central Sardinia. European Journal of Mineralogy. 1989;1: 657–666. doi:10.1127/ejm/1/5/0657

37. Valera RG, Valera PG, Rivoldini A. Sardinian ore deposits and metals in the Bronze Age. In: Lo Schiavo F, Giumlia-Mair A, Sanna U, Valera R, editors. Archaeometallurgy in Sardinia from the origin to the Early Iron Age. Montagnac: M. Mergoil; 2005. pp. 43–87.

38. Asael D, Matthews A, Bar-Matthews M, Harlavan Y, Segal I. Tracking redox controls and sources of sedimentary mineralization using copper and lead isotopes. Chemical Geology. 2012;310–311: 23–35. doi:https://doi.org/10.1016/j.chemgeo.2012.03.021

39. Ehrlich S, Butler I, Halicz L, Rickard D, Oldroyd A, Matthews A. Experimental study of the copper isotope fractionation between aqueous Cu(II) and covellite, CuS. Chemical Geology. 2004;209: 259–269. doi:https://doi.org/10.1016/j.chemgeo.2004.06.010

40. Harlavan Y, Bar-Matthews M, Matthews A, Asael D, Segal I. Tracing the sources of sedimentary Cu and Mn ores in the Cambrian Timna Formation, Israel using Pb and Sr isotopes. Journal of Geochemical Exploration. 2017;178: 67–82. doi:https://doi.org/10.1016/j.gexplo.2017.03.016

41. Hauptmann A. The archaeometallurgy of copper: Evidence from Faynan, Jordan. Berlin, Heidelberg: Springer; 2007.

42. Hauptmann A, Begemann F, Heitkemper E, Pernicka E, Schmitt-Strecker S. Early copper produced at Feinan, Wadi Araba, Jordan. Archeomaterials. 1992;6: 1–33.

43. Ketelaer A, Hauptmann A. In the shadow of Timna? The mining region of Wadi Amram: New analytical and archaeological aspects. METALLA. 2017;22: 169–183. doi:10.46586/metalla.v22.2016.i2.169-183

44. Segal I, Bar-Matthews M, Matthews A, Harlavan Y, Asael D. Provenance of ancient metallurgical artifacts: Implications of new Pb isotope data from Timna ores. In: Hauptmann A, Modaressi-Tehrani D, editors. Archaeometallurgy in Europe III: Proceedings of the 3rd International Conference, 29 June to 1 July 2011. Bochum: Deutsches Bergbau-Museum; 2015. pp. 221–228.

45. Atzeni C, Massidda L, Sanna U. Investigations and results. In: Lo Schiavo F, Giumlia-Mair A, Sanna U, Valera R, editors. Archaeometallurgy in Sardinia from the origin to the Early Iron Age. Montagnac: M. Mergoil; 2005. pp. 115–183.

46. Gale NH. Lead isotope studies: Sardinia and the Mediterranean. Provenance studies of artefacts found in Sardinia. Instrumentum. 2006;23: 29–34.

47. Montero Ruiz I, Manunza MR, Lo Schiavo F, Valera P, Gil Ibarguchi IJ, Rafael N, et al. The Funtana Coberta-Ballao Hoard: new copper provenances in Nuragic metallurgy. In: Giumlia-Mair A, Lo Schiavo F, editors. Bronze Age metallurgy on Mediterranean islands: In honour of Robert Maddin and Vassos Karageorgis. Drémil-Lafage: Editions Mergoil; 2018. pp. 139–164. Available: https://zenon.dainst.org/Record/001575060

48. Stos-Gale ZA, Gale NH, Bass GF, Pulak C, Galili E, Sharvit J. The copper and tin ingots of the Late Bronze Age Mediterranean. 1998. pp. 115–126.

49. Ridgway D, Ridgway FR, Macnamara E. The bronze hoard from S. Maria in Paulis, Sardinia. London: British Museum; 1984.

50. Balmuth MS, Tylecote RF. Ancient copper and bronze in Sardinia: Excavation and analysis. Journal of Field Archaeology. 1976;3: 195. doi:10.2307/529386

51. Riederer J. Metallanalysen sardischer Bronzen. In: Thimme J, editor. Kunst und Kultur Sardiniens: vom Neolithikum bis zum Ende d Nuraghenzeit: Ausstellung, Badisches Landesmuseum Karlsruhe im Karlsruher Schloss vom 18 Apr-13 Juli 1980, Museum für Vor- u Frühgeschichte d Staatlichen Museen Preussischer Kulturbesitz Berlin in Berlin-Charlottenburg vom 31 Juli-14 Sept 1980. Karlsruhe: Müller; 1980. pp. 156–160.

52. Cincotti A, Massidda L, Sanna U. Chemical and isotope characterization of lead finds at the Santa Barbara nuraghe (Bauladu, Sardinia). Journal of Cultural Heritage. 2003;4: 263–268. doi:https://doi.org/10.1016/j.culher.2003.06.002

53. Clemenza M, Billeci B, Carpinelli M, Ferrante M, Fiorini E, Gasperetti G, et al. Sant'Imbenia (Alghero): Further archaeometric evidence for an Iron Age market square. Archaeological and Anthropological Sciences. 2021;13: 181. doi:10.1007/s12520-021-01425-x
