# Supplementary material for: Multiproxy analysis unwraps origin and fabrication biographies of Sardinian figurines: On the trail of metal-driven interaction and mixing practices in the early first millennium BCE
Source: PLoS One. 2025 Sep 10;20(9):e0328268. doi: 10.1371/journal.pone.0328268 (PMC12422461; doi:10.1371/journal.pone.0328268)
Supplement: Attachment — Submitted filename: Response to reviewers.docx [file pone.0328268.s005.docx]

**In the following, we reply to the editor and reviewer directly after their comments.**

**We marked our comments with:** Comment of the authors: “…”

PONE-D-25-10968

Multiproxy analysis unwraps origin and fabrication biographies of Sardinian figurines: On the trail of metal-driven interaction and mixing practises in the early first millennium BCE

PLOS ONE

Dear Dr. Berger,

Thank you for submitting your manuscript to PLOS ONE. After careful consideration, we feel that it has merit but does not fully meet PLOS ONE’s publication criteria as it currently stands. Therefore, we invite you to submit a revised version of the manuscript that addresses the points raised during the review process.

Please submit your revised manuscript by Jun 07 2025 11:59PM. If you will need more time than this to complete your revisions, please reply to this message or contact the journal office at [plosone@plos.org](mailto:plosone@plos.org). When you're ready to submit your revision, log on to <https://www.editorialmanager.com/pone/> and select the 'Submissions Needing Revision' folder to locate your manuscript file.

Please include the following items when submitting your revised manuscript:

- A rebuttal letter that responds to each point raised by the academic editor and reviewer(s). You should upload this letter as a separate file labeled 'Response to Reviewers'.
- A marked-up copy of your manuscript that highlights changes made to the original version. You should upload this as a separate file labeled 'Revised Manuscript with Track Changes'.
- An unmarked version of your revised paper without tracked changes. You should upload this as a separate file labeled 'Manuscript'.

If you would like to make changes to your financial disclosure, please include your updated statement in your cover letter. Guidelines for resubmitting your figure files are available below the reviewer comments at the end of this letter.

If applicable, we recommend that you deposit your laboratory protocols in protocols.io to enhance the reproducibility of your results. Protocols.io assigns your protocol its own identifier (DOI) so that it can be cited independently in the future. For instructions see: [https://journals.plos.org/plosone/s/submission-guidelines#loc-laboratory-protocols](https://track.editorialmanager.com/CL0/https:%2F%2Fjournals.plos.org%2Fplosone%2Fs%2Fsubmission-guidelines%23loc-laboratory-protocols/1/010f019664f4dda1-c2887996-2e78-48a7-97bc-ae0ccdf2a1e8-000000/n9nMEkzije1an4K1XBjZUWpzwKaNyTFN1xJwOG6VaUk=208). Additionally, PLOS ONE offers an option for publishing peer-reviewed Lab Protocol articles, which describe protocols hosted on protocols.io. Read more information on sharing protocols at [https://plos.org/protocols?utm_medium=editorial-email&utm_source=authorletters&utm_campaign=protocols](https://track.editorialmanager.com/CL0/https:%2F%2Fplos.org%2Fprotocols%3Futm_medium=editorial-email%26utm_source=authorletters%26utm_campaign=protocols/1/010f019664f4dda1-c2887996-2e78-48a7-97bc-ae0ccdf2a1e8-000000/BO7asPMjDjrcCll-cKx8AlCy-LKQa6tZA69mTVCE40w=208).

We look forward to receiving your revised manuscript.

Kind regards,

Vanessa Forte, PhD
Academic Editor
PLOS ONE

Journal Requirements:

1. When submitting your revision, we need you to address these additional requirements.

Please ensure that your manuscript meets PLOS ONE's style requirements, including those for file naming. The PLOS ONE style templates can be found at

<https://journals.plos.org/plosone/s/file?id=wjVg/PLOSOne_formatting_sample_main_body.pdf> and

[https://journals.plos.org/plosone/s/file?id=ba62/PLOSOne_formatting_sample_title_authors_affiliations.pdf](https://track.editorialmanager.com/CL0/https:%2F%2Fjournals.plos.org%2Fplosone%2Fs%2Ffile%3Fid=ba62%2FPLOSOne_formatting_sample_title_authors_affiliations.pdf/1/010f019664f4dda1-c2887996-2e78-48a7-97bc-ae0ccdf2a1e8-000000/akpV4Y9O8dqa430URvMX0Wbascopw_YyF-NPJM4iRK8=208)

Comment of the authors: “Checked and, as far as we can assess it, our manuscript complies with PLOS ONE's style requirements.”

2. In your manuscript, please provide additional information regarding the specimens used in your study. Ensure that you have reported human remain specimen numbers and complete repository information, including museum name and geographic location.

If permits were required, please ensure that you have provided details for all permits that were obtained, including the full name of the issuing authority, and add the following statement:

'All necessary permits were obtained for the described study, which complied with all relevant regulations.'

Comment of the authors: “We have inserted the respective sentence in the manuscript in the chapter ‘Sample information’. The person who has permitted sampling is co-author of the paper (G Salis).”

If no permits were required, please include the following statement:

'No permits were required for the described study, which complied with all relevant regulations.'

For more information on PLOS ONE's requirements for paleontology and archeology research, see [https://journals.plos.org/plosone/s/submission-guidelines#loc-paleontology-and-archaeology-research](https://track.editorialmanager.com/CL0/https:%2F%2Fjournals.plos.org%2Fplosone%2Fs%2Fsubmission-guidelines%23loc-paleontology-and-archaeology-research/1/010f019664f4dda1-c2887996-2e78-48a7-97bc-ae0ccdf2a1e8-000000/DK05MeSE4ny8RFqVqeGDnNzu3i-AeaTgkkYKPn2Or10=208).

3. Thank you for stating the following in the Acknowledgments Section of your manuscript: [We are grateful to the Augustinus Foundation for funding the Metals & Giants project 2024–2027 (grant agreement 23–1869) and to the Danish Ministry of Culture FORM 2019 for funding analyses and the initial fieldwork at Matzanni, begun in 2019. Further funding for stable isotope analysis was obtained from the European Research Council through an Advanced Grant (grant agreement no. 323861). Osmium isotope analyses were made possible by funding resources from CEZA. The research presented is moreover indebted to Aarhus University Arts for funding V Matta’s PhD project, and to the research programme Material Culture and Heritage for covering costs of extra analyses. We are very grateful to Dr C Pilo and Dr A Usai for help during sampling in the archaeological storerooms of Cagliari and Sorradile (the Soprintendenza Archeologia, Belle Arti e Paesaggio per la Città Metropolitana di Cagliari e le Province di Oristano e Sud Sardegna). We thank B Höppner, G Brügmann, M Lockhoff and S Klaus for sample preparation and for performing the chemical analyses with ICP-Q-MS and isotopic measurements with MC-ICP-MS.]

We note that you have provided funding information that is not currently declared in your Funding Statement. However, funding information should not appear in the Acknowledgments section or other areas of your manuscript. We will only publish funding information present in the Funding Statement section of the online submission form.

Comment of the authors: “Deleted.”

Please remove any funding-related text from the manuscript and let us know how you would like to update your Funding Statement. Currently, your Funding Statement reads as follows: [Grant agreement 23–1869 to HV, MKH, GS. Augustinus Foundation funding the Metals & Giants project. <https://augustinusfonden.dk/en>

The foundation played no role in the study design, data collection and analysis, decision to publish, or preparation of the manuscript]

Please include your amended statements within your cover letter; we will change the online submission form on your behalf.

Comment of the authors: “We included the statement within the cover letter.”

4. We note that there is identifying data in the Supporting Information file <S2 Table.xlsx>. Due to the inclusion of these potentially identifying data, we have removed this file from your file inventory. Prior to sharing human research participant data, authors should consult with an ethics committee to ensure data are shared in accordance with participant consent and all applicable local laws.

Data sharing should never compromise participant privacy. It is therefore not appropriate to publicly share personally identifiable data on human research participants. The following are examples of data that should not be shared:

-Name, initials, physical address
-Ages more specific than whole numbers
-Internet protocol (IP) address
-Specific dates (birth dates, death dates, examination dates, etc.)
-Contact information such as phone number or email address
-Location data
-ID numbers that seem specific (long numbers, include initials, titled “Hospital ID”) rather than random (small numbers in numerical order)

Data that are not directly identifying may also be inappropriate to share, as in combination they can become identifying. For example, data collected from a small group of participants, vulnerable populations, or private groups should not be shared if they involve indirect identifiers (such as sex, ethnicity, location, etc.) that may risk the identification of study participants.

Additional guidance on preparing raw data for publication can be found in our Data Policy (<https://journals.plos.org/plosone/s/data-availability#loc-human-research-participant-data-and-other-sensitive-data>) and in the following article: <http://www.bmj.com/content/340/bmj.c181.long>.

Please remove or anonymize all personal information (ID), ensure that the data shared are in accordance with participant consent, and re-upload a fully anonymized data set. Please note that spreadsheet columns with personal information must be removed and not hidden as all hidden columns will appear in the published file.

Comment of the authors: “Removed.”

5. We note that Figures 1,3 and 19 in your submission contain [map/satellite] images which may be copyrighted. All PLOS content is published under the Creative Commons Attribution License (CC BY 4.0), which means that the manuscript, images, and Supporting Information files will be freely available online, and any third party is permitted to access, download, copy, distribute, and use these materials in any way, even commercially, with proper attribution. For these reasons, we cannot publish previously copyrighted maps or satellite images created using proprietary data, such as Google software (Google Maps, Street View, and Earth). For more information, see our copyright guidelines: <http://journals.plos.org/plosone/s/licenses-and-copyright>.

We require you to either (1) present written permission from the copyright holder to publish these figures specifically under the CC BY 4.0 license, or (2) remove the figures from your submission:

Comment of the authors: “Maps in Figs 1 and 3 have been produced Open Source QGIS software 3.20 geo reference data from this source: <https://www.sardegnageoportale.it/index.php?xsl=2420&s=40&v=9&c=14468&es=6603&na=1&n=10&esp=1&tb=14401>

Map in Fig 19 have been produced Open Source QGIS software 3.20 based based on the ESRI shaded relief map.

All data there is published under the CC BY 4.0 license.

No special license request is necessary.”

1. You may seek permission from the original copyright holder of Figures 1,3 and 19 to publish the content specifically under the CC BY 4.0 license.

We recommend that you contact the original copyright holder with the Content Permission Form (<http://journals.plos.org/plosone/s/file?id=7c09/content-permission-form.pdf>) and the following text:

“I request permission for the open-access journal PLOS ONE to publish XXX under the Creative Commons Attribution License (CCAL) CC BY 4.0 (<http://creativecommons.org/licenses/by/4.0/>). Please be aware that this license allows unrestricted use and distribution, even commercially, by third parties. Please reply and provide explicit written permission to publish XXX under a CC BY license and complete the attached form.”

Please upload the completed Content Permission Form or other proof of granted permissions as an ""Other"" file with your submission.

In the figure caption of the copyrighted figure, please include the following text: “Reprinted from [ref] under a CC BY license, with permission from [name of publisher], original copyright [original copyright year].”

Comment of the authors: “We added such a phrase in the respective captions.”

2. If you are unable to obtain permission from the original copyright holder to publish these figures under the CC BY 4.0 license or if the copyright holder’s requirements are incompatible with the CC BY 4.0 license, please either i) remove the figure or ii) supply a replacement figure that complies with the CC BY 4.0 license. Please check copyright information on all replacement figures and update the figure caption with source information. If applicable, please specify in the figure caption text when a figure is similar but not identical to the original image and is therefore for illustrative purposes only.

The following resources for replacing copyrighted map figures may be helpful:

USGS National Map Viewer (public domain): <http://viewer.nationalmap.gov/viewer/>

The Gateway to Astronaut Photography of Earth (public domain): <http://eol.jsc.nasa.gov/sseop/clickmap/>

Maps at the CIA (public domain): <https://www.cia.gov/library/publications/the-world-factbook/index.html> and <https://www.cia.gov/library/publications/cia-maps-publications/index.html>

NASA Earth Observatory (public domain): <http://earthobservatory.nasa.gov/>

Landsat: <http://landsat.visibleearth.nasa.gov/>

USGS EROS (Earth Resources Observatory and Science (EROS) Center) (public domain): [http://eros.usgs.gov/#](http://eros.usgs.gov/)

Natural Earth (public domain): [http://www.naturalearthdata.com/](http://track.editorialmanager.com/CL0/http:%2F%2Fwww.naturalearthdata.com%2F/1/010f019664f4dda1-c2887996-2e78-48a7-97bc-ae0ccdf2a1e8-000000/8dk4wCD5gsofpqjoCnt7GU-PnGDDJ2M9fyjLjIAldD8=208)

6. Please review your reference list to ensure that it is complete and correct. If you have cited papers that have been retracted, please include the rationale for doing so in the manuscript text, or remove these references and replace them with relevant current references. Any changes to the reference list should be mentioned in the rebuttal letter that accompanies your revised manuscript. If you need to cite a retracted article, indicate the article’s retracted status in the References list and also include a citation and full reference for the retraction notice.

Comment of the authors: “Checked. It is complete and correct.”

Additional Editor CommentS:

Thank you for your submission and patience in receiving the reviews. The referees were all very positive regarding the paper and suggested minor revisions. I therefore return the paper to you under the category of minor revisions to give you the chance to reflect on the reviewer comments and make any necessary changes prior to final acceptance of the paper. Thank you for choosing Plos One.


[Note: HTML markup is below. Please do not edit.]

Reviewers' comments:

Reviewer's Responses to Questions

**Comments to the Reviewers**

Comment of the authors: “Dear reviewers, thanks for your time and valuable input regarding our manuscript. We hope to have addressed all the points raised appropriately. In the following we reply to your points directly after your lines.”

**Comments to the Author**

1. Is the manuscript technically sound, and do the data support the conclusions?

The manuscript must describe a technically sound piece of scientific research with data that supports the conclusions. Experiments must have been conducted rigorously, with appropriate controls, replication, and sample sizes. The conclusions must be drawn appropriately based on the data presented.

Reviewer #1: Yes

Reviewer #2: Yes

2. Has the statistical analysis been performed appropriately and rigorously?

Reviewer #1: Yes

Reviewer #2: Yes

3. Have the authors made all data underlying the findings in their manuscript fully available?

The [PLOS Data policy](http://track.editorialmanager.com/CL0/http:%2F%2Fwww.plosone.org%2Fstatic%2Fpolicies.action%23sharing/1/010f019664f4dda1-c2887996-2e78-48a7-97bc-ae0ccdf2a1e8-000000/hXOAry6oS1FUsQ0eRzutEjk8Rlh4vCnwggFJaoVD85A=208) requires authors to make all data underlying the findings described in their manuscript fully available without restriction, with rare exception (please refer to the Data Availability Statement in the manuscript PDF file). The data should be provided as part of the manuscript or its supporting information, or deposited to a public repository. For example, in addition to summary statistics, the data points behind means, medians and variance measures should be available. If there are restrictions on publicly sharing data—e.g. participant privacy or use of data from a third party—those must be specified.

Reviewer #1: Yes

Reviewer #2: Yes

4. Is the manuscript presented in an intelligible fashion and written in standard English?

PLOS ONE does not copyedit accepted manuscripts, so the language in submitted articles must be clear, correct, and unambiguous. Any typographical or grammatical errors should be corrected at revision, so please note any specific errors here.

Reviewer #1: Yes

Reviewer #2: Yes

5. Review Comments to the Author

Please use the space provided to explain your answers to the questions above. You may also include additional comments for the author, including concerns about dual publication, research ethics, or publication ethics. (Please upload your review as an attachment if it exceeds 20,000 characters)

Reviewer #1: The study presents important new data that inform long-standing questions in the study of LBA - EIA Sardinia / central & eastern Mediterranean, metallurgical production and technological / communication networks. As such, the paper is a very welcome addition to the existing literature. I particularly enjoyed the suppl. mat. on Cypriot copper, which answers many of my questions that formed from reading the paper. I think a comment on the potential mixing of copper could / should be added in the main text, particularly considering recent results reported from the Balkans.

Comment of the authors: “There was already a comment in the main text addressing the potential mixing of Cypriot copper. We hope that this is sufficient?”

Although I am happy with the paper in its present form, i would like to invite the authors to a few considerations:

A comment on the possibility of a correlation between the reported mixing lines / ores and the typology / form / function of the analysed objects (I understand that they are mostly fragmens) in the text would be welcome.

Comment of the authors: “In the Materials and Methods section, we described that only fragments of bronzetti—primarily arms and feet of statuettes—were analysed. As far as can be determined based on stylistic criteria, all fragments belong to the Uta-Abini style. More detailed typological classifications are not possible. Nevertheless, we have added clarifying remarks in the relevant sections to indicate that both the arms and feet fall along the same mixing lines.”

Furthermore, I have some comments on figure 10: Fig. 10 only works if the population within each histogram is somehow normalized and, thus, made comparable to its lake-mirror histogram. As of now, any differences in the distribution could result from sampling bias. This would also fix the frequency issue in the Pb histograms. Importantly, all x axes need ticks and some indication of the binning / scale.

Comment of the authors: “Thanks for this helpful comment! We revised the figure and used relative frequencies instead of absolute. Also, we inserted numbers and ticks for the x-axes.”

In a few occasions, where % with five decimals are give, possibly ppm values would be best reported.

Comment of the authors: “In order to ensure consistency within the respective columns of element concentrations we would like to keep %, even though we agree that too many decimals can be confusing.”

Finally, I wonder if the long tables with the results should be best included in an xl file as suppl. mat. This would increase the flow / readability of the text and also make the data more easily reusable by future studies. Which leads me to ask: are these tables already duplicated in the 1st worksheet of S2 Table xl file?

Comment of the authors: “We can follow this objection. And yes, the data in these tables is reproduced in Supplement S2. We would like to leave it for now as it stands, but if the editor agrees with your comment, the tables in the main text can be deleted.”

Considering the above, I am very happy to see this paper published.

Reviewer #2: This is a technical analysis Sardinian metalwork dating to the early 1st millennium BC from the Nuragic sanctuaries of Santa Vittoria, Su Monte, and Abini. The two key aspects that stand out in terms of the significance of the study are the sophistication of the analytical approach to complex questions of metal provenance and mixing and the relative lack of comparable metallurgical analyses on the iconic object category - the iconic bronzetti. Given the central role of Sardinia in the Mediterranean metals trade in the early 1st millennium BC (and earlier) and the potential for sources spanning the Levant to Iberia, establishing the source of metals and the extent/nature of mixing/recycling is a challenge.

The paper is systematic and detailed in the analysis of the samples and admirably cautious in its interpretations. It nonetheless highlights persuasively that the copper is likely from the Sa Duchessa mine in Sardinia and the Alcudia valley or the Linares district in Iberia. However, the most useful contribution is the osmium isotope signature that demonstrates clearly an approach and result that shows the exploitation of Sardinian copper sources by local communities for the bronzetti. This should lay to rest a long running debate due to issues with lead isotope analyses and lead contamination and will be very useful for researchers in future. There is also a strong and useful analytical point in this assemblage on the lack of exploitation of Sardinian tin - another long running debate. The results evaluating mixing are certainly interesting though unsurprisingly not as definitive. It is however clear that Cypriot copper was not involved.

The authors are very well positioned indeed to lead this research and draw extensively and impressively on a wide range of scholarship, including their own recent and internationally significant research. They obtained the requisite permissions for sampling which should also be commended.

However, there needs to be a greater clarity and narrative flow for the reader in this paper which requires the authors to step back and consider what they want to say to the broader audience with this excellent paper. Otherwise there is a real potential for losing the reader. Currently, the introduction highlights that the paper addresses the debates over the local production of metal on Sardinia with the Cypriot alternative as a hypothesis. The analysis then not only addresses the debates on provenance - and highlights Iberia but goes into detail on questions of mixing/recycling/production sequences. The discussion addresses both of these and then rather abruptly provides a summary societal/landscape sanctuary model based on the very early results of the regional Sardinian project that the authors recently started. It is not always clear where the discussions over/references to Cyprus, the Levant, Iberia or elsewhere fit in and given that the main contribution is on Sardinia in the metals trade then this needs to be expanded and clarified. It currently feels if different sections where written by different authors with overlapping but slightly different purposes in mind for the paper.

Comment of the authors: “We carefully considered the concerns raised and reviewed the manuscript multiple times. We agree with some of your objections to a certain extent. To address your points, we revised the introduction, substantially restructured the results section to improve readability, and added subheadings to the discussion section to make the argumentation more accessible. Additionally, we made improvements and additions throughout all sections of the manuscript.”

6. PLOS authors have the option to publish the peer review history of their article ([what does this mean?](https://track.editorialmanager.com/CL0/https:%2F%2Fjournals.plos.org%2Fplosone%2Fs%2Feditorial-and-peer-review-process%23loc-peer-review-history/1/010f019664f4dda1-c2887996-2e78-48a7-97bc-ae0ccdf2a1e8-000000/WwsQ0vCTONBn7-W8dl9zfsKPPfKAUZLX2i0vDrVl9Q8=208)). If published, this will include your full peer review and any attached files.


If you choose “no”, your identity will remain anonymous but your review may still be made public.

Comment of the authors: “The authors choose “yes”

**Do you want your identity to be public for this peer review?** For information about this choice, including consent withdrawal, please see our [Privacy Policy](https://track.editorialmanager.com/CL0/https:%2F%2Fwww.plos.org%2Fprivacy-policy/1/010f019664f4dda1-c2887996-2e78-48a7-97bc-ae0ccdf2a1e8-000000/jPxu9DSLmXtBAmzT-007xHWkYwZfJlLLcWXbuj2FTuM=208).

Reviewer #1: No

Reviewer #2: No

[NOTE: If reviewer comments were submitted as an attachment file, they will be attached to this email and accessible via the submission site. Please log into your account, locate the manuscript record, and check for the action link "View Attachments". If this link does not appear, there are no attachment files.]

While revising your submission, please upload your figure files to the Preflight Analysis and Conversion Engine (PACE) digital diagnostic tool, [https://pacev2.apexcovantage.com/](https://track.editorialmanager.com/CL0/https:%2F%2Fpacev2.apexcovantage.com%2F/1/010f019664f4dda1-c2887996-2e78-48a7-97bc-ae0ccdf2a1e8-000000/Y3L3Ss2R1itbOWew4w471KNI7WiClbnV5oqTF9SMCbg=208). PACE helps ensure that figures meet PLOS requirements. To use PACE, you must first register as a user. Registration is free. Then, login and navigate to the UPLOAD tab, where you will find detailed instructions on how to use the tool. If you encounter any issues or have any questions when using PACE, please email PLOS at [figures@plos.org](mailto:figures@plos.org). Please note that Supporting Information files do not need this step.

*In compliance with data protection regulations, you may request that we remove your personal registration details at any time.* [*(Remove my information/details)*](https://track.editorialmanager.com/CL0/https:%2F%2Fwww.editorialmanager.com%2Fpone%2Flogin.asp%3Fa=r/1/010f019664f4dda1-c2887996-2e78-48a7-97bc-ae0ccdf2a1e8-000000/ebQ8Smr4nVj0kELITAbJoWuE6zqIP1eCxs9fKNwsoos=208)*. Please contact the publication office if you have any questions.*
